# Assessment of High-Risk Human Papillomavirus Infections Using Clinician- and Self-Collected Cervical Sampling Methods in Rural Women from Far Western Nepal

**DOI:** 10.1371/journal.pone.0101255

**Published:** 2014-06-30

**Authors:** Derek C. Johnson, Madhav P. Bhatta, Jennifer S. Smith, Mirjam-Colette Kempf, Thomas R. Broker, Sten H. Vermund, Eric Chamot, Shilu Aryal, Pema Lhaki, Sadeep Shrestha

**Affiliations:** 1 Department of Epidemiology, University of Alabama at Birmingham, Birmingham, Alabama, United States of America; 2 College of Public Health, Kent State University, Kent, Ohio, United States of America; 3 Department of Epidemiology, University of North Carolina, Chapel Hill, North Carolina, United States of America; 4 School of Nursing, University of Alabama at Birmingham, Birmingham, Alabama, United States of America; 5 Department of Health Behavior, University of Alabama at Birmingham, Birmingham, Alabama, United States of America; 6 Department of Biochemistry and Molecular Genetics, University of Alabama at Birmingham, Birmingham, Alabama, United States of America; 7 Vanderbilt University, Institute for Global Health, Nashville, Tennessee, United States of America; 8 Nepal Family Health Division, Kathmandu, Nepal; 9 Nepal Fertility Care Center, Kathmandu, Nepal; Georgetown University, United States of America

## Abstract

**Introduction:**

Nepal has one of the highest cervical cancer rates in South Asia. Only a few studies in populations from urban areas have investigated type specific distribution of human papillomavirus (HPV) in Nepali women. Data on high-risk HPV (HR-HPV) types are not currently available for rural populations in Nepal. We aimed to assess the distribution of HR- HPV among rural Nepali women while assessing self-collected and clinician-collected cervico-vaginal specimens as sample collection methods for HPV screening.

**Methods:**

Study participants were recruited during a health camp conducted by Nepal Fertility Care Center in Achham District of rural far western Nepal. Women of reproductive age completed a socio-demographic and clinical questionnaire, and provided two specimens; one cervical-vaginal specimen using a self-collection method and another cervical specimen collected by health camp auxiliary nurse midwives during a pelvic examination. All samples were tested for 14 different HR-HPV mRNA and also specific for HPV16/18/45 mRNA.

**Results:**

Of 261 women with both clinician- and self-collected cervical samples, 25 tested positive for HR-HPV, resulting in an overall HR-HPV prevalence of 9.6% (95% confidence Interval [CI]: 6.3–13.8). The overall Kappa value assessing agreement between clinician- and self-collected tests was 0.62 (95% CI: 0.43–0.81), indicating a “good” level of agreement. Abnormal cytology was reported for 8 women. One woman identified with squamous cell carcinoma (SCC), and 7 women with high grade squamous intraepithelial lesions (HSIL). Seven of the 8 women tested positive for HR-HPV (87.5%) in clinician-collected samples and 6 in self-collected samples (75.0%).

**Conclusion:**

This is the first study to assess HR-HPV among rural Nepali women. Self-collected sampling methods should be the subject of additional research in Nepal for screening HR-HPV, associated with pre-cancer lesions and cancer, in women in rural areas with limited access to health services.

## Introduction

The World Health Organization (WHO) Information Centre on Human Papillomavirus (HPV) and Cervical Cancer states that the incidence of cervical cancer in Nepal is 24.2 per 100,000, making Nepal a country with one of the highest cervical cancer rates in South Asia [1]. Over 2100 cases of cervical cancer are reported in Nepal each year, with a case-fatality rate of over 50%. However, this estimate is most likely an underestimation of the actual cases due to lack of national cancer registry as well as screening and follow-up [1], [2]. Epidemiologic and virologic data indicate that oncogenic HPVs are the primary (and necessary) causal agents of cervical cancer [2], [3]. Worldwide HPV16 and HPV18/45 account for over 70% of the high risk HPV strains (HR-HPV) associated with cervical cancer. While it is assumed HR-HPV types follow similar distributional patterns in invasive cervical cancer across the globe, the actual distribution of HPV is not known for several resource-limited countries, including Nepal [4]. Previous studies estimate the prevalence of HR-HPV types in other South Asian countries to be between 8–14% in the general female population [5], [6]. However, currently, there are no official WHO estimates of the prevalence or type-specific distribution of HPV in representative population-based samples of women in Nepal.

To date, information on HPV in Nepal has been limited to study samples drawn from populations in selected urban areas in the central region of the country, with an emphasis on women with invasive cervical cancer [7], [8]. An International Agency for Research on Cancer (IARC) study reported a prevalence of 8.6%, 6.1%, and 1.9% for any HPV, HR-HPV types, and HPV16, respectively among 932 married women aged 15–59 years from a general population in Bharatpur, a city in the south-central part of Nepal [7]. Among a smaller subset of women diagnosed with invasive cervical cancer, Bhusal and colleagues found that HPV16 was the most common HR-HPV (50% of HR-HPV) detected followed by HPV18 (18% of HR-HPV) [8]; however, this result from 44 cancer patients cannot be generalizable. The distribution of HPV in the general population including cervical cancer patients in various parts of the country is still unknown.

There is considerable variation in the limited number of cervico-vaginal and cervical cancer reports across Nepal, with most reports originating from hospital-based registries because there is no national cancer registry [9]–[12]. The far western region of Nepal is very rural with limited access to health services made worse by extreme topography, economic, socio-cultural, and ethno-linguistics regional differences. This area is known to have a higher prevalence of risk factors for poor reproductive health compared to the general Nepali population, which may exacerbate the rates of HPV infections and cervical cancer in the region [5], [13]. Only 52% of women in far western Nepal give birth in the presence of a skilled birth attendant compared to the national average of 82% [14], [15]. The infant mortality rate in Achham is 61 per 1,000 and the maternal mortality rate is 950 per 100,000, both of which are higher than the national average of 42 per 1,000 and 170 per 100,000, respectively [14]. A significant proportion of the adult male population in far western Nepal seasonally migrate to cities in India to work as migrant laborers, which could put them at an increased risk for sexually transmitted infections (STIs) including HPV [16].

The introduction of self-sampling for HPV detection in Nepal could help mitigate the problems of insufficient access to cervical screening. HPV self sampling has been shown to have high rates of agreement with physician collected HPV samples [17], [18] and in most settings self collection has demonstrated a better ability to detect cervical abnormalities of CIN2+ or greater than Pap smear testing in women with low socioeconomic status [19]. Providing a self sampling option for women living in less developed countries could also bolster screening participation by making screening more convenient and private for women [20].

The regional differences in the risk profile for HPV infections and cervical cancer within Nepal necessitate the need for assessing the prevalence and type-specific distribution of HR-HPV infections in different regions of the country. Currently, there are no estimates of HPV prevalence or distribution in Nepal’s rural populations. This is the first study to assess the distribution of HR-HPV and the type-specific distribution of HR-HPV16/18/45 among women in rural far western Nepal. Our study also compares the self-collection of cervico-vaginal specimens with clinician-collected specimens as a method of sample collection for HPV screening in a resource-limited setting.

## Materials and Methods

### Ethics Statement

The Institutional Review Board at the University of Alabama at Birmingham and Ethics Review Board at the Nepal Health Research Council approved this study.

### Study site and population

This study was conducted in the Sanphebagar Village Development Committee (VDC) within the Achham District. Achham is one of the most remote districts in far western Nepal. Due to poor and mountainous road conditions, it takes over 13 hours to drive the 500 km from Kathmandu to Mangalsen, the Achham district headquarter. Achham district covers an area of 1,680 km^2^ and only 36 of its 75 VDCs are connected by roads. According to the 2011 Nepal Demographic Health Survey, the population of females in Achham District was 141,643, which included 34,204 adolescent girls (aged 10–19), 60,937 women of reproductive age (aged 15–49) and 21,143 seniors (aged 60 years and above) [13]. The district’s population experiences a greater burden of risk factors for poor health outcomes, has higher rates of seasonal migration and access to a fewer health care facilities compared to the general Nepali population [21]. Currently, only two hospitals operate in Achham District; the government’s district hospital in Mangalsen and Bayalpata Hospital, operated by non-profit organization Nyaya Health in collaboration with the Nepali government [13]. Achham district is also served by 12 health posts and several temporary primary health care outreach clinics primarily conducted by various non-governmental organizations (NGOs).

Women of reproductive age were recruited from the Sanphebagar VDC, approximately 25 km from Mangalsen, during a one day health camp conducted on July 5^th^, 2013 by Nepal Fertility Care Center (NFCC). Established in 1988, NFCC is a Nepali NGO with the primary objective of complementing and supplementing the Government of Nepal’s national reproductive health programs [22]. Health camps are regularly conducted by NFCC and advertised ahead of time through networking with government bodies at all levels of administration from the center to the VDC level. Women who attended the health camp received a wide array of routine free reproductive health services including family planning counseling and sexually transmitted infection (STI) testing. Trained Auxiliary Nurse Midwives (ANMs) with several years of clinical experience performed pelvic examinations while volunteer health professionals experienced in conducting surveys assisted in recruitment coordination and data collection during the health camp. Women were included in the study if they were of reproductive age (at least 16 but no more than 60 years old) and had a cervix but not menstruating and not pregnant during the visit. Consent forms were distributed and read to each participant before they agreed to enroll in the study. Each form was signed and dated by the study participant, a witness, and the study coordinator. A woman who opted not to participate in the research study was provided the same clinical services during the health camp as those participating in the research study.

### Survey Instrument Development and Administration

A questionnaire to assess socioeconomic, clinical, reproductive health, and migration related factors was developed for this study. The questionnaire was first developed in English, then translated into Nepali, and then translated back to English independently for quality control. NFCC staff assisted in the development of the questionnaire in order to insure that the survey questions were culturally sensitive and appropriate. In order to help assure the validity and comprehensiveness of the survey instrument, questions were based on English translations of the Demographic and Health Survey [23], and the National Living Standards Survey (NLSS) [24].

A dialect of the Nepali language known as “Dotyali” is spoken in far western Nepal. However, the term “Dotyali” is a macro-term used to describe a wide variety of local dialects in far western Nepal [25]. The local Dotyali/Nepali dialect spoken in Achham does not have a written form; thus, making it difficult to translate the questionnaire into the local dialect. People living in Achham, however, speak and understand Nepali. In order to ensure that participants fully understood each question and were comfortable while completing the survey, female interviewers who spoke the local Achham dialect were recruited locally.

### Biospecimen Collection and Laboratory Analyses

Each study participant was asked to provide two specimens during her health camp visit. One specimen was self-collected, using the APTIMA Cervical Specimen Collection and Transport (CSCT) kit (Hologic/Gen-Probe, San Diego, CA), and another specimen was collected by a health camp ANM during pelvic examination. While waiting for their pelvic examination, women were given instructions on self-collection of a cervico-vaginal sample. A health staff member, centrally trained to give instructions on self-sampling procedures, instructed the women to insert a cytobrush (Hologic/Gen-Probe, San Diego, CA) into the vagina as far as possible, rotate it 5 times in each direction, then swirl the cytobrush in the APTIMA specimen transport medium (Hologic/Gen-Probe, San Diego, CA). Health camp ANMs collected clinician cervical specimens by inserting a cytobrush into the cervical canal and rotating 3–5 times, withdrawing and then rotating firmly around the full circumference of the transformation zone. Cytobrushes were then swirled in ThinPrep (TP) PreservCyt medium (Hologic/Gen-Probe, San Diego, CA).

Both clinician- and self-collected cervico-vaginal samples were transported to the Hologic/Gen-Probe, Inc. laboratory in San Diego for HPV testing. Laboratory testing of HPV was performed using a generic APTIMA HR-HPV mRNA (APTIMA HPV) (Hologic/Gen-Probe, San Diego, CA) and then specific genotyping using APTIMA HPV16 18/45 Genotype (Hologic/Gen-Probe, San Diego, CA) Assays [26]. Samples were first tested using the APTIMA HPV Assay to detect the presence of E6/E7 mRNA from at least one of 14 different types of HR-HPV (HPV 16, 18, 31, 33, 35, 39, 45, 51, 52, 56, 58, 59, 66, and 68). Samples testing positive by APTIMA HPV Assays were retested with APTIMA HPV16 18/45 Genotype Assay to detect the presence of HPV16 or HPV18/45 genotypes. Cervical cytology was assessed for research purposes using clinician-collected ThinPrep PreservCyt medium (Hologic/Gen-Probe, San Diego, CA), with results classified according to the Bethesda criteria. Women with atypical squamous cells of undetermined significance (ASCUS) or worse were referred to either the government’s district hospital in Mangalsen or Bayalpata Hospital for additional testing and follow-up.

### Statistical analysis

The mean, standard deviation, median, inter-quartile range, and percentages were calculated for self-reported demographic, socioeconomic, and behavioral characteristics. An exact version of McNemar chi-square test was used to test for differences in HPV prevalence in clinician vs. self-collected specimens. Positive and negative agreement percentages and Kappa statistics values were used to describe agreement in detecting HR-HPV between clinician- vs. self-collected samples. We report point estimates for prevalence, % concordance, and Kappa statistics and associated 95% confidence intervals (95% CI). SAS version 9.2 (SAS Institute, Cary, NC) was used to perform all statistical analyses.

## Results

**Figure 1:**
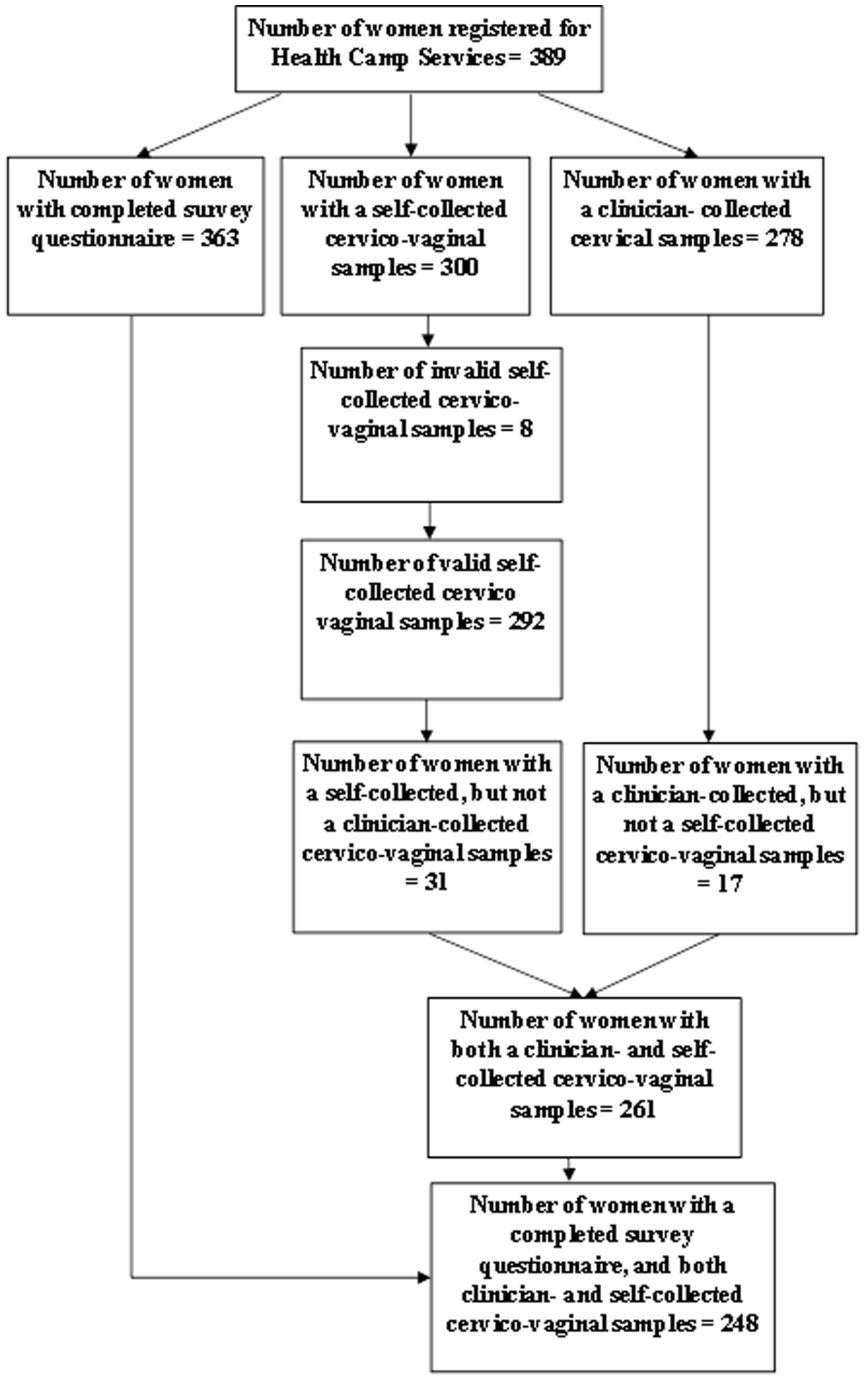
Study Sample Size Algorithm according to Strengthening the Reporting of Observational Studies in Epidemiology (STROBE) Guidelines.

In total, 389 women registered for the health camp. Three hundred and sixty three women completed a survey; clinician-collected specimens were available from 278 women while self-collected specimens were available from 300 women (Figure 1). Eight of the 300 self-collected samples were excluded due to invalid HPV test results. Thirty one women with a self-collected specimen did not have a clinician-collected specimen; 17 women with a clinician-collected specimen did not have a self-collected specimen. In all, 261 women had both self- and clinician-collected valid samples, with self-reported questionnaires being available for 248 of these women (Figure 1).

The median age of the participants was 33 years (IQR: [26–40]) and the median age at first marriage was 17.5 years (IQR 15–19). The median number of children per woman was 3.0 (IQR: [2.0–4.0]) and 56% of women currently used some form of contraception. Over half of the study participants reported having heard of cervical cancer (58.1%), while only 16% reported having heard of HPV (Table 1).

**Table 1:** Socio-demographic and Behavioral Characteristics, and Human Papillomavirus (HPV) mRNA Results in Women Participating in a Health Camp in Achham District of the Far western Nepal.

Characteristics	Mean (SD), median (IQR) or Frequency (%)§
**Positive for HR-HPV** * **(n = 261)**	
Self-Collected Sample	20 (7.7)
Clinician-Collected Sample	17 (6.5)
Either Sample**	25 (9.6)
**Age in years**	
Mean (SD)	33.8 (8.8)
Median (IQR)	33 (26–40)
**Age at first marriage (n = 240)**	
Mean (SD)	17.3 (2.6)
Median (IQR)	17.5 (15–19)
**Number of Children (n = 248)**	
Mean (SD)	3.2 (1.6)
Median (IQR)	3.0 (2.0–4.0)
**Husband ever migrated for work (n = 240)**	
Yes	121 (50.4)
No	119 (49.6)
**Heard of cervical cancer (n = 246)**	
Yes	143 (58.1)
No	103 (41.9)
**Heard of HPV (n = 223)**	
Yes	36 (16.1)
No	187 (83.9)
**Currently use contraception (n = 234)**	
Yes	132 (56.4)
No	102 (43.6)

§ SD = Standard Deviation; IQR = Inter-quartile range.

*High Risk HPV (HR-HPV) defined as testing positive for one of the following genotypes: (16, 18, 31, 33, 35, 39, 45, 51, 52, 56, 58, 59, 66, and 68).

**Tested positive for HR-HPV in either clinician- or self-collected biospecimen.

Twenty women tested positive for HPV in clinician-collected samples while 17 women tested positive for HPV in self-collected samples and 25 out of 261 individuals tested positive for HR-HPV in either sampling method, resulting in an over-all HR-HPV prevalence of 9.6% (95% CI 6.3–13.8). Five individuals who tested positive for HR-HPV tested positive for HPV16, resulting in an overall prevalence of 1.9% (5 HPV16+/261 individuals with both a clinician- and self-collected test) for HPV16. Only one individual tested positive for HPV18/45. Thus, among those with HR-HPV, only 24% (6/25) were either type HPV16 or HPV18/45. Five individuals tested positive for HR-HPV in the self-collected specimens, but did not test positive for HR-HPV in the clinician-collected samples. Eight individuals tested positive in clinician-collected samples tests but not in the self-collected samples.

The non-stratified Kappa value assessing agreement between the HR-HPV test results using clinician- and self-collected specimens was 0.62 (95% CI 0.43–0.81), indicating a “good” level of agreement (Table 2). The overall negative agreement was 94.7% (95% CI 93.3–96.2) and overall positive agreement was 48.0% (95% CI 30.1–65.9) (Table 2). There was 100% agreement between self-collected and clinician-collected sample test results for women who tested positive for HPV16. The Kappa value for women who tested positive for HR-HPV other than HPV16 or HPV 18/45 was 0.49 (95% CI 0.26–0.73). The negative agreement for women who tested positive for HR-HPV other than HPV16 or HPV 18/45 was 94.9% (95% CI 93.5–96.3) and the positive agreement 35.0% (95% CI 12.1–57.9) (Table 3).

**Table 2:** Prevalence and Concordance of High Risk-Human Papillomavirus (HR-HPV) Test Results Between Clinician-collected and Self-collected Cervico-vaginal Samples at a Health Camp in Achham District, Nepal in 261 women.

	Prevalence		Concordance		
	Clinician-collected sample (95% CI)§	Self-collectedsample (95%_CI)§	% Negative agreement (95%CI)§	% Positive agreement (95%CI)§	Kappa (95%CI)§
**HR-HPV** * **(n = 25)**	7.7 (4.4–10.9)	6.5 (4.1–10.2)	94.7 (93.3–96.2)	48.0 (30.1–65.9)	0.62 (0.43–0.81)
**HPV16 Only (n = 5)**	1.9 (0.9–3.5)	1.9 (0.9–3.5)	100.0 (100.0–100.0)	100.0 (100.0–100.0)	1.00 (1.0–1.0)
**HR-HPV not including HPV16 (n = 20)**	5.8 (3.0–9.1)	4.6 (2.7–7.9)	94.9 (93.5–96.3)	35.0 (12.1–57.9)	0.49 (0.26–0.73)
**HR-HPV in women** **testing positive for** **HSIL or SCC** ** **(n = 8)**	87.5 (46.7–99.3)	75.0 (35.6–95.5)	N/A	N/A	N/A

§ 95% CI = 95% Confidence Interval;

*High-Risk HPV (HR-HPV) defined as testing positive for one of the following genotypes: (16, 18, 31, 33, 35, 39, 45, 51, 52, 56, 58, 59, 66, 68);

**HSIL = High-grade Squamous Intraepithelial Lesion; SCC = Squamous Cell Carcinoma.

**Table 3:** High-risk Human Papillomavirus (HR-HPV) Test* Results on Clinician-collected or Self-collected Cervico-vaginal Specimens Stratified by Liquid-based Cytology, Achham District, Nepal (N = 278).

	Liquid-based Cytology** Results§ on Clinician-collected Samples								
	SCC	HSIL	ASC-H	LSIL	AGUS	ASCUS	UNSAT	WNL/BCC/ACTINO	Total
**Total Cytology Results**	1	7	5	4	2	32	16	211	278
**HR-HPV (Clinician-collected Specimen)**	1	6	1	3	0	2	1	6	20
Type 16/18/45	1	3	0	0	0	0	0	2	6
Other HR-HPV	0	3	1	3	0	2	1	4	14
**HR-HPV (Self-collected Specimen)**	1	5	0	2	0	3	1	5	17
Type 16/18/45	1	3	0	0	0	0	0	2	5
Other HR-HPV	0	2	0	2	0	3	1	3	12

*APTIMA HR-HPV mRNA Assay (Hologic/Gen-Probe, San Diego, CA).

**ThinPrep PreservCyt medium ((Hologic/Gen-Probe, San Diego, CA).

§ SCC = Squamous Cell Carcinoma; HSIL = High-grade Squamous Intraepithelial Lesion; ASC-H = Atypical Squamous Cell-cannot exclude HSIL; AGUS = Atypical Glandular Cells of Undetermined Significance; LSIL = Low-grade Squamous Intraepithelial Lesion; ASCUS = Atypical Squamous Cells of Undetermined Significance; UNSAT = Unsatisfactory; WNL = Within Normal Limits; BCC = benign cellular changes; ACTINO = Actinomycosis.

ThinPrep (TP) (Hologic/Gen-Probe, San Diego, CA) cervical cytology readings based on clinician-collected samples were available for 278 women. High grade cytological abnormality was reported for 8 women, with one woman identified with squamous cell carcinoma (SCC), and 7 women with high grade squamous intraepithelial lesions (HSIL). Of the 8 women with either SCC or HSIL, 7 of them tested positive for HR-HPV (87.5%) in clinician-collected samples and 6 in self-collected samples (75%). However, 4 of the 7 women who tested positive for HR-HPV tested positive for HPV16 or HPV 18/45 in clinician-collected samples versus 3 in the self-collected samples (Table 3). Sixteen ThinPrep (TP) (Hologic/Gen-Probe, San Diego, CA) samples were unable to be tested for cytology due to inadequate amount of specimen from spillage or evaporation during shipment or other sampling issues.

## Discussion

To the best of our knowledge, this is the first HR-HPV prevalence study conducted among rural women from far western Nepal. Of 261 women with both clinician- and self-collected cervico-vaginal specimens who were tested for 14 HR-HPV types, 25 women tested positive with an overall HR-HPV prevalence of 9.6% (95% CI 6.3–13.8). A non-stratified Kappa value of 0.62 indicated “good” agreement between self- and clinician-collected HPV samples. The cytology of 8 women indicated either SCC or HSIL, of which 7 women who had clinician-collected samples tested positive for HR-HPV and 6 women who had self-collected samples tested positive for HR-HPV.

Our study’s prevalence of HR-HPV (9.6%) is higher than the prevalence of HR-HPV (6.1%) found in Bharatpur, Nepal [7] and is similar to the prevalence of HR-HPV (9.9%) in India’s northern state of Uttar Pradesh, which borders central and western Nepal [27]. In each of these studies, HPV16 was the most common type of HR-HPV, while HPV18/45 was less common. While our study was limited to testing only HPV16 18 and/or 45 genotypes, it could still detect the presence of 14 different HR-HPV, without specific genotypes. Our findings suggest the distribution of HR-HPV follows the general HPV distribution patterns found throughout South Asia [28] and is similar to the distribution of HR-HPV in most regions of the world [28], [29].

Only one woman tested positive for HPV18/45 (the APTIMA HPV 16 18/45 genotype assay can differentiate HPV 16 from HPV 18 and/or HPV 45, but does not differentiate between HPV 18 and HPV 45), yet HPV genotypes 18 and 45 represented approximately 20% of HPV strains linked to cervical abnormalities in studies conducted by IARC and Bhusal [7], [8]. While the proportion of women testing positive for HPV18/45 in our study is slightly lower than the world-wide distribution of HPV18/45 [28], [29], it is likely that our study’s lower prevalence rates of HPV16 and HPV18/45 are due to small sample size and not attributable to a different distribution of HPV16 or HPV18/45.

The number of women with an abnormal cytology reading was low. However, these abnormalities pose a significant health risk to women given the dearth of reproductive health care access in rural Nepal. Despite the official Nepali government protocol of cervical screening using visual inspection with acetic acid (VIA), a paltry number of women actually undergo cervical screening each year [30]. Without proper access to reproductive health care, minor complications could result in bigger complications over time due to a lack of access to health care. Therefore, it is important to advise women with even minor cervical abnormalities on the importance of proper follow-up.

Non-stratified kappa values assessing the testing agreement between clinician- and self-collected specimens was 0.62 (95% CI 0.43–0.81), indicating a good level of agreement between the two sampling methods. Several studies conducted in rural areas of developing countries which have limited access to cervical screening have also found the results of HPV testing using self-collected samples comparable to the results of HPV testing using clinician-collected samples [18], [31], [32]. Other studies have suggested that HPV DNA tests in self-collected samples as a primary screening method for cervical cancer is superior to the IARC recommended visual inspection with acetic acid method of cervical screening [18], [33].

Only a few studies have assessed the utility of testing for HPV using self-collected samples in less-developed countries [34]–[36]. The majority of these studies detect the presence of HR-HPV DNA, the HPV virus genome [34], while only a few studies have utilized HR-HPV messenger RNA (mRNA) tests, which detect the expression of genes related to E6/E7 [36]. Previous clinical studies suggest that testing for HR-HPV E6/E7 mRNA using the APTIMA HPV assay results in similar sensitivity and higher specificity for detecting high-grade lesions compared to DNA based tests [37], [38]. However, the HPV prevalence as measured by the APTIMA HPV assay may be lower as compared to prevalence determined by a DNA based test without impacting clinical sensitivity for detection of cervical disease. This could result in underestimation of the actual prevalence of HPV in the population.

This cross-sectional study could not assess lifetime exposure to HPV, nor could it measure HPV persistence, which precedes and predicts the development of cervical precancerous lesions. However, while our study was not longitudinal, HPV prevalence is correlated with the risk of developing cervical cancer [39] and can still remain a good surrogate, specifically in a resource-limited setting like Nepal with limited data on HPV infection and cervical cancer. Our study’s small sample size hinders the precision of our prevalence and concordance estimates, which is indicated by the size of the confidence intervals. However, we were able to collect a wide array of data in this remote region in Nepal, which included both clinician- and self-collected samples in addition to a survey on demographic, socioeconomic, and behavioral characteristics. Our study sample may not be representative of the far western region of Nepal and may not be generalizable to the entire district of Achham. There are 9 districts in the far western Development Region of Nepal, covering the three major ecological zones (Mountains, Hills, and Terai). Achham District lies in the ecological “Hills” zone which is geographically and ethnically different from the other districts/ecological zones in the far western region. Therefore, it is difficult to assess any selection bias during recruitment of participants.

In summary, our study assessed the prevalence of HR-HPV among women in a rural region of far western Nepal. Furthermore, our study demonstrated that the self-collected cervical sample for HPV testing is a viable method with a high level of concordance with clinician-collected cervical samples. Self-sampling, while not as sensitive as clinician-collected specimens for detecting prevalent HR-HPV, associated with cervical pre-cancer or cancer [18], should be the subject of additional research as a method for screening women in rural areas with limited access to health services.
